# Supplementary material for: Adsorption energy as a metric for wettability at the nanoscale
Source: Sci Rep. 2017 Apr 11;7:46317. doi: 10.1038/srep46317 (PMC5387734; doi:10.1038/srep46317)
Supplement: Supplementary Information [file srep46317-s1.pdf]

Adsorption energy as a metric for wettability at the nanoscale

Ronaldo Giro[1], Peter W. Bryant[1], Michael Engel[1,2], Rodrigo F. Neumann[1] & Mathias B. Steiner[1,2]

[1] *IBM Research, Av. Pasteur 138/146, CEP 22290-240, Rio de Janeiro, RJ, Brazil*

[2] *IBM Research, 1101 Kitchawan Rd, Yorktown Heights, NY 10598, United States of America.*

Supplementary Methodology

All-Atom Molecular Dynamics (AAMD) We simulated in three dimensions the behavior of a decane droplet surrounded by nitrogen gas over a glass surface at the molecular scale by using AAMD.

A set of cubic boxes composed of decane molecules and surrounded by nitrogen molecules was placed onto a passivated glass slab (see Table S1 for details). The molecules were distributed randomly in defined regions of the simulation box, as shown in Fig. S1, and packaged by using the Packmol package[1].

The dimensions of the simulation boxes for the system consisting of glass, decane, and nitrogen, are shown in Table S1. The dimensions labeled L were chosen in order to isolate the interactions between periodic images. Periodic boundary conditions were used in all directions. The number of decane and nitrogen molecules, as well as the volume of the simulation box, were

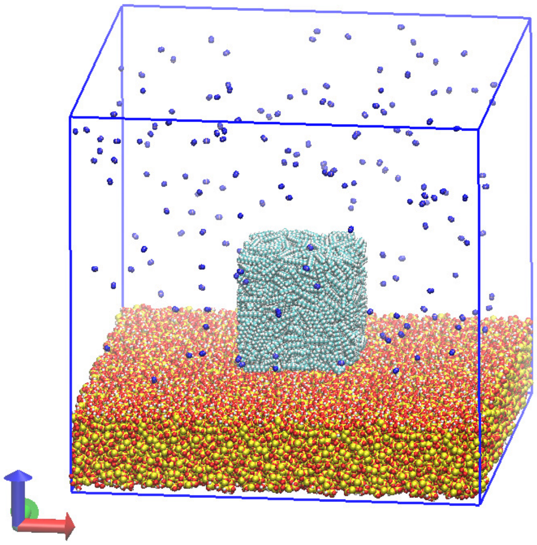

Supplemental Material, Figure S1: (a) 3D representation of the input configuration of decane molecules (light blue) packed in a cubic arrangement over a glass surface (yellown and red) and surrounded by nitrogen molecules (dark blue).

chosen in order to reproduce the equilibrated density of pure nitrogen and pure decane, obtained by following the protocol for a specific temperature and pressure: a 1 ps run was performed for the NVE ensemble (micro canonical ensemble - constant Number of atoms, Volume, and Energy), followed by 10 ps for the NVT ensemble (canonical ensemble - constant Number of atoms, Volume, and Temperature), and finally 2 ns for the NPT ensemble (isothermal-isobaric or grand canonical ensemble - constant Number of atoms, Pressure, and Temperature). The protocols were performed for a cubic box (10 nm edge) containing 3089 decane molecules, and for a cubic box (20 nm edge) containing 160 nitrogen molecules. In both cases periodic boundary conditions were used in all three dimensions. The preliminary CMD simulations were performed first for decane and the isolated nitrogen systems, because the density of the glass/decane/nitrogen system cannot be equilibrated using the NPT ensemble, as the NPT ensemble does not contain enough nitrogen

molecules to transfer energy from collisions (equivalent to pressure) to the glass surface. As a consequence, in the case of the glass/decane/nitrogen system, we performed CMD simulations with the NVT ensemble only.

Table S1: Input box sizes and number of molecules considered in AAMD simulations.

decane cubic box side	# of decane molecules	# of nitrogen molecules	System box size L_x (nm)	L_y (nm)	L_z (nm)
6.0	670	180	23	23	21.7
6.1	704	180	23	23	21.7
6.2	739	180	23	23	21.7
6.3	776	180	23	23	21.7
6.4	813	180	23	23	21.7
7.0	1045	370	28.5	28.5	25.6
9.0	2257	316	28.5	28.5	29.5
13.0	6815	1030	40.1	40.1	34.5

Coarse-Grained Molecular Dynamics (CGMD) In CGMD a set of atoms is represented as a single "super-atom," which we will refer to as CG bead. The most intuitive procedure is to represent CG beads as the center of mass of a set of atoms. For glass we define two types of beads: surface beads and bulk beads. A bulk bead represents three Silicon and six Oxygen atoms, while a surface bead represents three Silicon, six Oxygen, and two Hydrogen atoms. Decane molecules are represented as three beads, and Nitrogen molecules as one single bead. The coarse-grained glass surface was built with three layers of bulk glass beads, and two layers, one on top and one on the bottom, arranged in a deformed cubic lattice (see Fig. S2 for details). In Table S2 we show the lattice vectors and the All-Atom unit cell coordinates for surface and bulk glass CG beads.

The CG interaction potential was built following a mapping procedure from AAMD. Due to difficulties in mapping amorphous materials such as glass, we considered α-quartz in (100)

Table S2: Lattice vectors and the All-Atom unit cell coordinates for surface and bulk glass CG beads. Dimensions in Angstroms.

primitive lattice vectors

	$\hat{x}$	$\hat{y}$	$\hat{z}$
$\vec{a}$	5.400	0.000	0.000
$\vec{b}$	0.000	4.910	0.000
$\vec{c}$	0.000	-2.455	4.252

atomic coordinates - CG unit cell

	x	y	z
	2.281	1.761	1.796

atomic coordinates - AA surface unit cell

chemical element	x	y	z
Si	0.000	0.000	0.000
Si	3.601	1.086	2.208
Si	1.800	3.541	2.045
O	1.171	4.028	0.639
O	2.430	2.081	1.763
O	2.972	-0.374	2.489
O	4.772	0.975	1.124
O	4.230	1.573	3.614
O	0.629	3.430	3.128
H	4.230	1.573	4.514
H	0.629	3.430	4.028

atomic coordinates - AA bulk unit cell

chemical element	x	y	z
Si	0.000	0.000	0.000
Si	3.601	1.086	2.208
Si	1.800	3.541	2.045
O	1.171	4.028	0.639
O	2.430	2.081	1.763
O	2.972	-0.374	2.489
O	4.772	0.975	1.124
O	4.230	1.573	3.614
O	0.629	3.430	3.128

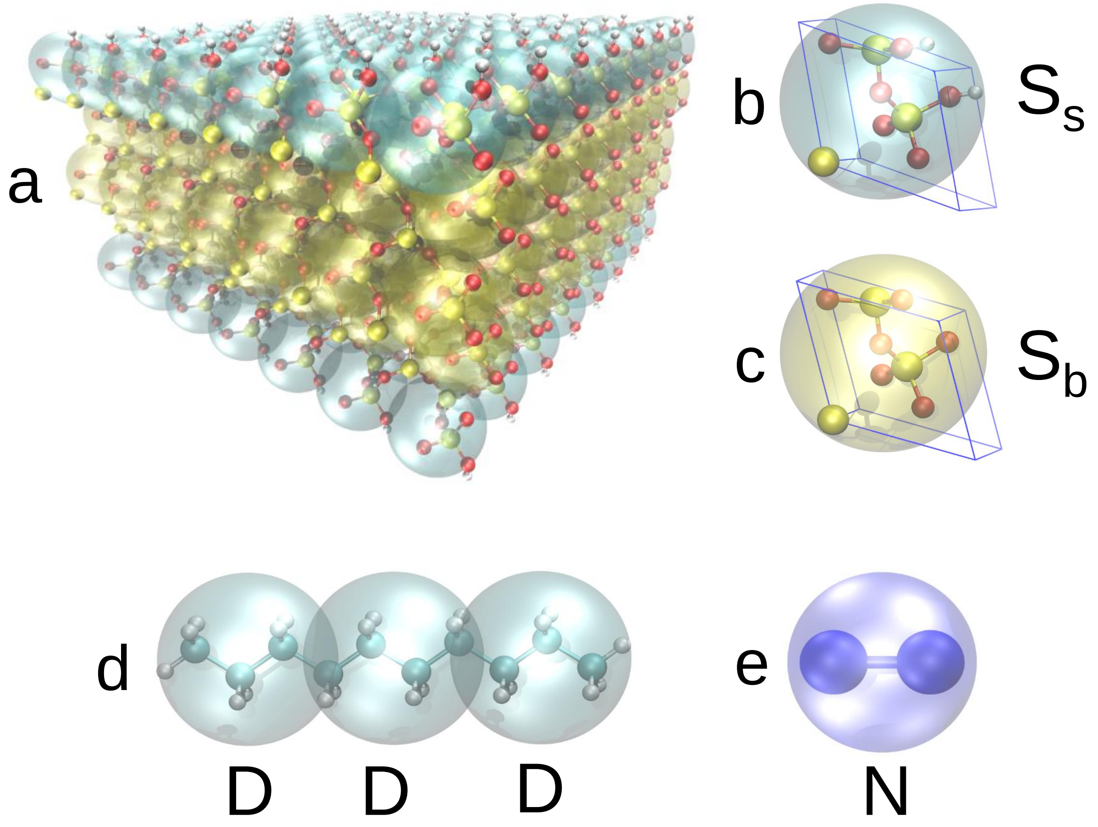

Supplemental Material, Figure S2: Coarse-grained (transparent) and All-Atom representation of (a) glass surface with three bulk layers (in transparent yellow) and two surface layers (in transparent light blue). (b) and (c) are the unit cells of a surface and a bulk bead. (d) and (e) are the CG representations of decane and nitrogen, respectively. In the All-Atom representation, Silicon atoms are in yellow, Oxygen in red, Hydrogen in white, Carbon in light blue, and Nitrogen in dark blue. The capital letters, S_b, S_s, D and N, label the coarse-grained beads types.

orientation. We expect this simplification to be a good approximation because: (i) The glass surface beads represent an average interaction of three Silicon, six Oxygen, and 2 Hydrogen atoms mapped from α-quartz AAMD. This average over a set of atoms results in a certain indistinguishability among the atoms' arrangements (amorphous in glass and crystalline in α-quartz). (ii) The glass surface beads mapped from All-Atom α-quartz shows the same concentration of hydroxyl (OH) as glass, i.e around 5 hydroxyls per nm^2. Thus the same surface chemistry as glass, is characterized.

By mapping α-quartz from All-Atom to the CG representation we extracted the bond length and bond angle potentials for glass, and the CG nonbonded potentials to describe the interactions between Nitrogen and decane with glass surface beads. In the present case, the systematic iterative Boltzmann inversion (IBI) numerical scheme from Fukuda *et al.*[2] was adopted. In this method, effective pair potentials between CG beads are determined from AAMD with an iterative refinement procedure.

The bond length potentials are represented by a harmonic potential:

$$U_{\text{bond}}(R) = K_{\text{bond}}(R - R_0)^2 \tag{S1}$$

In Table S3 we show the parameters for silica (glass) and decane. The decane parameters are extracted from the MARTINI Force Field[3].

Table S3: Bond length potential parameters for glass and decane beads.

bonds between bead types	K_{bond} (Kcal/molÅ^2)	R_0 (Å)
$S_b - S_b$	125.0	5.13
$S_b - S_b$	105.0	5.64
$S_b - S_s$	75.0	5.13
$S_s - S_s$	105.0	5.13
$S_s - S_s$	47.0	5.64
D – D[a]	1.4938	4.7

[a] parameters from the MARTINI Force Field[3]

For glass beads, the bond angle potential is represented by a harmonic potential:

$$U_{\text{angle1}}(\theta) = K_{\text{angle1}}(\theta - \theta_0)^2 \tag{S2}$$

According to Marrink *et al.*[3], the bond angle potential for decane beads is represented as a cosine-squared potential:

$$U_{\text{angle2}}(\theta) = K_{\text{angle2}}[\cos(\theta) - \cos(\theta_0)]^2 \tag{S3}$$

In Table S4 we show the parameters for bond the angle potential for glass and decane beads.

Table S4: Bond angle potential parameters for glass and decane beads.

angles between bead types	K_{angle1} (Kcal/mol radian^2)	θ_0 (degrees)	Potential
$S_b - S_b - S_b$	30.0	180	harmonic – eq. S2
$S_b - S_b - S_b$	19.6	60	harmonic – eq. S2
$S_b - S_b - S_b$	47.6	90	harmonic – eq. S2
$S_b - S_b - S_s$	15.0	60	harmonic – eq. S2
$S_b - S_b - S_s$	50.2	90	harmonic – eq. S2
$S_s - S_b - S_s$	14.5	60	harmonic – eq. S2
$S_s - S_s - S_s$	36.6	90	harmonic – eq. S2
$S_s - S_s - S_s$	16.0	180	harmonic – eq. S2
angles between beads types	K_{angle2} (Kcal/mol)	θ_0 (degrees)	Potential
D–D–D[a]	2.9876	180	cosine squared – eq. S3

[a] parameters from the MARTINI Force Field[3]

To describe the nonbonded interactions between Nitrogen-Nitrogen, Decane-Surface glass beads, and Nitrogen-Surface glass beads, a shifted Lenard-Jones (LJ) 12-6 potential energy function was employed as an initial guess for the IBI method:

$$U_{\text{LJ}}(r) = 4\epsilon_{ij}\left[\left(\frac{\sigma_{ij}}{r}\right)^{12} - \left(\frac{\sigma_{ij}}{r}\right)^{6}\right] \tag{S4}$$

We used the same procedure as described in the work of Fukuda *et al.*[2], to obtain the parameters σ_{ij} and ϵ_{ij} for the initial guess LJ 12-6 potential. We observed a good agreement between the AAMD and CGMD radial distribution functions and the numerical density profiles for Nitrogen-Nitrogen and Nitrogen-Surface glass beads nonbonded interactions, respectively. Given the good agreement there was no need to proceed with IBI refinement. Thus the nonbonded interactions between these beads are described simply by the LJ 12-6 potential from eq. S4.

For Decane-Decane beads, the nonbonded interactions are described by the LJ 12-6 potential, as in eq. S4, and the paramenters were obtained from the MARTINI Force Field[3].

In Table S5 we show the σ_{ij} and ϵ_{ij} parameters for nonbonded interactions between Nitrogen-Nitrogen, Nitrogen-Surface glass beads, and Decane-Decane beads.

Table S5: LJ 12-6 potential parameters to describe the unbonded interactions between Nitrogen-Nitrogen, Nitrogen-Surface glass beads, and Decane-Decane beads.

unbonded interactions between bead types	ϵ_{ij} (Kcal/mol)	σ_{ij} (Å)	cutoff (Å)
S_s – N	0.57880	4.987	12.0
D – D[a]	0.83596	4.700	12.0
D – N	0.41083	4.200	12.0
N – N	0.20190	3.700	12.0

[a] parameters from the MARTINI Force Field[3]

On the other hand, for nonbonded interactions between Decane-Surface glass beads, a good agreement between the numerical density profile from AAMD and CGMD was not observed. Thus the IBI refinement procedure, as described in Fukuda *et al.*[2], was performed. After the IBI refinement, a good agreement between AAMD and CGMD numerical was obtained (see Fig. S3(a)).

In Fig. S3(b) we show the Potential of Mean Force obtained from IBI refinement. We observe a small deviation from the initial guess LJ 12-6 potential in the region from 10 to 15 Å.

To build the Coarse-Grained system composed of decane over a glass surface and surrounded by nitrogen, the same procedure as described in AAMD section was followed. The dimensions of the simulation boxes are shown in Table S6.

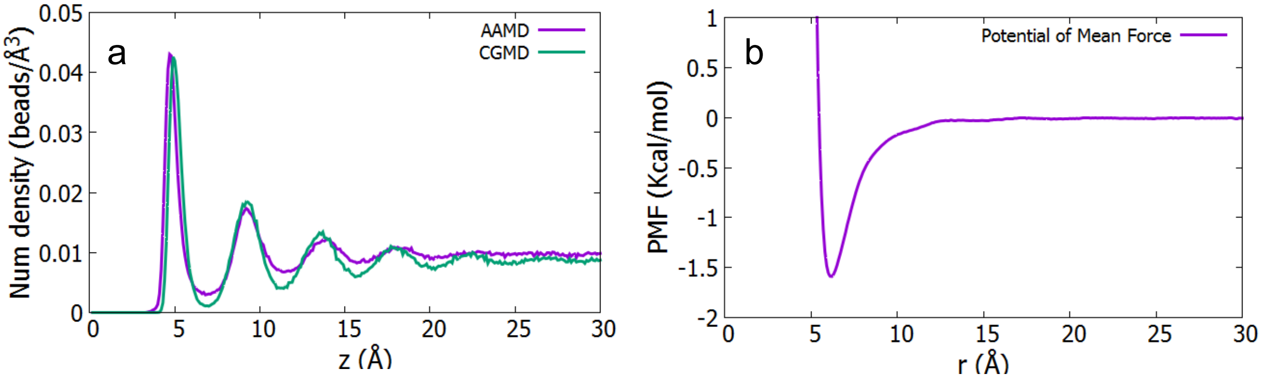

Supplemental Material, Figure S3: (a) Numerical density profile for decane beads of the glass surface. The numerical density profiles are calculated across the interface between glass and decane fluid. (b) Potential of Mean Force obtained after the IBI refinement procedure.

Table S6: Input box sizes and number of molecules considered in CGMD simulations.

decane cubic box side	# of decane molecules	# of nitrogen molecules	System box size		
			L_x (nm)	L_y (nm)	L_z (nm)
15.0	10450	54800	156.8	157.0	106.0
30.0	83400	54250	156.8	157.0	106.0
57.0	591657	50365	156.8	157.0	106.0
113.5	4519850	404600	312.9	313.4	201.3

Substrate preparation In this study, we use float glass slides having a size of 20x20mm^2 and a thickness of 170 μm (Paul Marienfeld GmbH&Co. KG). In a first step, we clean the glass substrates by sequential ultrasonication in ultrapure Isopropanol and Acetone. Secondly, substrates are dry cleaned using an oxygen plasma for 20min.

Droplet generation As a model oil we use Decane (anhydrous, $\geq$99%, Sigma-Aldrich Co. LLC) to prepare micro- and nanoscale size droplets as analyzed in the main manuscript. We prepare droplets by ultrasonic vaporization (Atomizer, Sonics Materials, Inc). Before each vaporization the ultrasonic nozzle has been cleaned by sequentially injecting isopropanol and acetone.

Measurements setup For AFM measurements, we used a DI Dimension 3000 (Veeco, now Bruker Corporation) operated in tapping mode using sharp silicon tips having an average radius of 10 nm (TESPA-V2, Bruker Corporation).

In similar experimental setups, it is common to raise questions concerning the volatility of Decane and the presence of contaminants. In that case small droplets would represent droplets of non-volatile contaminants left over after the complete evaporation of the decane droplets. With regards to the volatility of Decane, while it is true that Decane's low vapor pressure allows it to evaporate rather quickly, the evaporation rate is still slow in comparison to the measurement time. This allows us to acquire high resolution AFM images of small Decane droplets before evaporation occurs (see Fig.S4 (b)). For example, droplet cross sections that are analyzed in the main manuscript are based on line scans. These line scans have been acquired in ca. 50 ms for the chosen AFM scan parameters which leads us to believe that evaporation is negligible factor.

Regarding the impact of contaminants, special care has been taken during sample preparation and droplet formation, as mentioned above. Reference AFM measurements on bare substrates show an atomically flat and clean surface (see Fig.S4 (a)). Further we observed after deposition an atomically-flat surface turn into a distribution of droplets of various sizes, spanning several orders of magnitude, which seems inconsistent with the contaminant hypothesis.

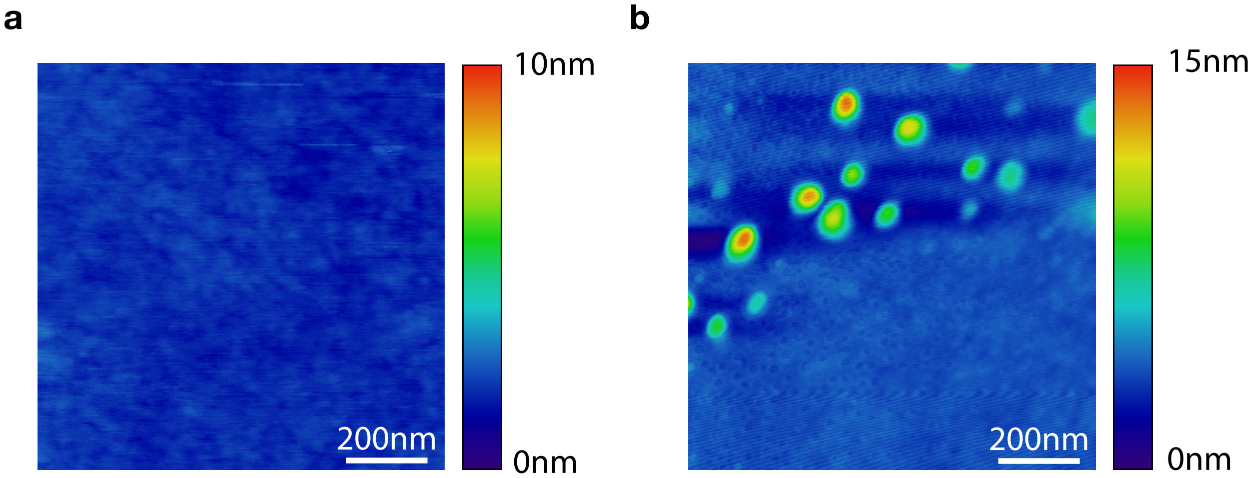

Supplemental Material, Figure S4: a) AFM image of a bare substrate prior to droplet deposition. b) AFM image of a sample after deposition of decane showing nanoscale droplets.

1. Martínez, L., Andrade, R., Birgin, E. G. & Martínez, J. M. PACKMOL: A package for building initial configurations for molecular dynamics simulations. *J. Comput. Chem.* **30**, 2157–2164 (2009). URL `http://dx.doi.org/10.1002/jcc.21224`.

2. Fukuda, M., Zhang, H., Ishiguro, T., Fukuzawa, K. & Itoh, S. Structure-based coarse-graining for inhomogeneous liquid polymer systems. *The Journal of Chemical Physics* **139**, 054901 (2013). URL `http://dx.doi.org/10.1063/1.4817192`.

3. Marrink, S. J., Risselada, H. J., Yefimov, S., Tieleman, D. P. & de Vries, A. H. The MARTINI

force field: coarse grained model for biomolecular simulations. *The Journal of Physical Chemistry B* **111**, 7812–7824 (2007). URL `http://dx.doi.org/10.1021/jp071097f`.
